# A Potential Role for Shed Soluble Major Histocompatibility Class I Molecules as Modulators of Neurite Outgrowth

**DOI:** 10.1371/journal.pone.0018439

**Published:** 2011-03-31

**Authors:** Lorraine R. Washburn, Dan Zekzer, Shoshana Eitan, Yuxin Lu, Hoa Dang, Blake Middleton, Christopher J. Evans, Jide Tian, Daniel L. Kaufman

**Affiliations:** 1 Department of Molecular and Medical Pharmacology, University of California Los Angeles, Los Angeles, California, United States of America; 2 Department of Psychiatry and Biobehavioral Sciences, University of California Los Angeles, Los Angeles, California, United States of America; University of North Dakota, United States of America

## Abstract

The neurobiological activities of classical major histocompatibility class I (MHCI) molecules are just beginning to be explored. To further examine MHCI's actions during the formation of neuronal connections, we cultured embryonic mouse retina explants a short distance from wildtype thalamic explants, or thalami from transgenic mice (termed “NSE-D^b^”) whose neurons express higher levels of MHCI. While retina neurites extended to form connections with wildtype thalami, we were surprised to find that retina neurite outgrowth was very stunted in regions proximal to NSE-D^b^ thalamic explants, suggesting that a diffusible factor from these thalami inhibited retina neurite outgrowth. It has been long known that MHCI-expressing cells release soluble forms of MHCI (sMHCI) due to the shedding of intact MHCI molecules, as well as the alternative exon splicing of its heavy chain or the action proteases which cleave off it's transmembrane anchor. We show that the diffusible inhibitory factor from the NSE-D^b^ thalami is sMHCI. We also show that COS cells programmed to express murine MHCI release sMHCI that inhibits neurite outgrowth from nearby neurons *in vitro*. The neuroinhibitory effect of sMHCI could be blocked by lowering cAMP levels, suggesting that the neuronal MHCI receptor's signaling mechanism involves a cyclic nucleotide-dependent pathway. Our results suggest that MHCI may not only have neurobiological activity in its membrane-bound form, it may also influence local neurons as a soluble molecule. We discuss the involvement of complement proteins in generating sMHCI and new theoretical models of MHCI's biological activities in the nervous system.

## Introduction

Classical major histocompatability complex class I (MHCI) Ia molecules play a central role in immune surveillance. The MHCI complex is tripartite, consisting of a heavy chain (≈44 kDa), β2 microglobulin (β2M) light chain (≈12 kDa) and a short polypeptide of 8–10 amino acids from a cytosolic antigen that is bound to a groove in the heavy chain [1], [2]. By presenting peptides from intracellular proteins on the cell surface, MHCI displays the proteins that are being produced within the cell to the immune system. MHCI on the cell surface is screened by CD8^−^ T cells using T cell receptors that were generated by gene rearrangement to identify cells expressing foreign antigens. Additionally, the level of MHCI expression on the cell surface is monitored by natural killer (NK) cells using germ line encoded MHCI receptors in order to identify cells that may be infected or transformed [2], [3], [4].

Although MHCI is well known as a membrane–bound molecule, soluble forms of MHCI (sMHCI) also exist which were first identified in the blood in the 1970's ([5], [6], reviewed in [7], [8]). sMHCI is generated by 1) the shedding of intact MHCI molecules (which contain the full-length heavy chain with its transmembrane segment and cytoplasmic tail [9], [10]); 2) an alternative RNA splicing pathway which deletes the heavy chain's exon 5 which encodes its transmembrane domain [11], [12], and 3) by the action of intracellular or extracellular proteases which cleave the heavy chain from its transmembrane anchor [7], [8], [10]. However, sMHCI has no known biological function under natural physiological conditions.

There is a growing recognition that molecules that have key roles in immune surveillance also have important roles in neurodevelopment. MHCI-deficient mice have an exuberance of retinal projections in their thalamus and enhanced long term potentiation, indicating that MHCI participates in activity-dependent synaptic elimination [13]. Additionally, PirB, which is a MHCI receptor of the innate immune system, is also a receptor for myelin inhibitors and PirB-deficient mice have aberrations in cortical neuroplasticity [14], [15]. Mice lacking the proteins involved in the classical complement cascade, C1q or C3, have deficiencies in CNS synapse elimination that are similar to those of MHCI-deficient mice, suggesting that MHCI-mediated synaptic elimination is dependent in part on the action of complement proteins [16].

We have been interested in classical MHCI's effects on wildtype neurons. We observed that picomolar levels of recombinant soluble MHCI monomers could inhibit neurite outgrowth from embryonic mouse retina explants in culture [17]. Interestingly, neurons were more sensitive to growth inhibition by self-MHCI than non-self MHCI monomers, regardless of the nature of the peptide presented. Neurons from MHCI-deficient mice were insensitive to growth inhibition by exogenous recombinant MHCI. These findings suggest that endogenous expression of MHCI is necessary to “educate” neurons to recognize self-MHCI, similar to the educational process that NK cells undergo to express MHCI receptors appropriate for the MHCI haplotype of the animal.

Recently, we studied transgenic C57Bl/6 mice (“NSE-D^b^” mice) which have a neuron-specific enolase promoter driving the expression of a full-length D^b^ heavy chain (matching their endogenous H-2D allele) specifically in their CNS neurons [18], [19]. These mice have elevated levels of functional D^b^ on the surface of their CNS neurons. We observed that enhanced neuronal MHCI expression led to abnormalities in neurodevelopment, including a smaller retina projection area in their thalamus, reduced synaptic makers and neurons in some regions of their hippocampus and reduced axonal sprouting responses after a hippocampal lesion [19]. Thus, elevated neuronal MHCI expression can lead to alterations in CNS morphology, synaptic connections and axon outgrowth.

In the current study, we wanted to further examine how ectopic neuronal MHCI expression affects interactions between neurons *in vitro*. As a model system, we used embryonic retina-thalamic explant co-cultures. MHCI is expressed at low levels in embryonic mouse thalami and its expression is greatly upregulated postnatally during the period of activity-dependent remodeling [13]. Normally, when embryonic retina explants are cultured near to an embryonic thalamus the retina ganglion cells (RGCs) extend axons that form arbors and functional connections within the thalamic explant [20], [21]. We were surprised to find that retinas placed near to a NSE-D^b^ thalamic explant had very stunted neurite outgrowth from the area proximal to the thalamic explant, suggesting that a diffusible factor from the NSE-D^b^ thalami inhibited retina neurite outgrowth at a distance.

We surmised that the diffusible factor was sMHCI. In the following studies, we demonstrate that conformationally correct sMHCI is released from NSE-D^b^ thalami, as well as COS cells programmed to express a full-length D^b^ heavy chain, leading to inhibition of neurite outgrowth from nearby neurons. Our results suggest that MHCI may not only have neurobiological activity as a membrane-bound molecule, it may also affect local neurons as a soluble molecule *in vivo*. We show that the neuroinhibitory activity of sMHCI can be blocked by a cAMP antagonist, suggesting that the neuronal MHCI receptor's signaling mechanism involves a cyclic nucleotide-dependent pathway. Finally, we discuss the role of complement proteins in producing sMHCI and new models of MHCI's biological activities in the nervous system.

## Materials and Methods

### Animals

All studies were approved by the UCLA Chancellor's Animal Research Committee (approval #2000-023-33). C57Bl/6 mice were purchased from The Jackson Laboratory (Bar Harbor, Maine). The generation of C57BL/6 NSE-D^b^ mice which express elevated levels of D^b^ on their neurons has been previously described [18], [19]. These transgenic mice have a neuron-specific enolase (NSE) promoter driving the expression of a D^b^ minigene (containing all the D^b^ coding region/exons) specifically in neurons, resulting in higher levels of MHCI on the cell surface of CNS neurons. The NSE-D^b^ mice were backcrossed with C57BL/6 mice for 10 generations and then bred them to homozygosity for the transgene [19].

### Retina-thalamic co-culture

Embryonic day 14 (E14) embryos from wildtype C57BL/6 or transgenic C57Bl/6 NSE-D^b^ mice were placed into petri dishes containing Leibovitz's L-15 media (Gibco BRL) and the retinas and thalami were isolated. A retina was then placed ≈0.5 mm from a wildtype, or a NSE-D^b^, thalamus in a matrigel matrix in an 8 chamber slide on ice. The slides were then incubated at 37°C for 30–60 minutes to solidify the gel. Neurobasal medium was added to each chamber (0.2 ml) and the co-cultures were coded and incubated for 4–5 days. The experiment was terminated by replacing the media with 4% PFA and storing the slides at 4°C. The co-cultures were scored in a subjective manner by two blinded individuals for the relative extent of retinal neurite outgrowth towards the thalamus (on a scale of 0–100). Only co-cultures with a distance of 0.5–1.0 mm between the tissues were analyzed. Duplicates of each group were tested simultaneously in at least 4 separate experiments. The scores of positive control cultures with wildtype thalamus were normalized to represent 100% outgrowth and all other group scores were adjusted accordingly.

### Antibody neutralization of MHCI

All antibodies were coded and added to co-cultures of wildtype retinas and NSE-D^b^ thalami in a blinded fashion. Mouse anti-D^b^ mAb (HB-27, ATCC designation 28-14-8S), control isotype-matched anti-K^k^ mAb (HB-16, ATCC designation 16-1-11N), anti-GAD65 mAb (GAD-6, Sigma-Aldrich), or mouse IgG2a (Sigma-Aldrich), all at 0.002 mg/ml, were added to the matrigel (prior to its solidification) and to the media. All these antibodies are the IgG2a isotype. Each experiment contained positive control co-cultures of wildtype retinas confronted with wildtype thalami. The co-cultures were incubated, fixed and scored in a blinded fashion, as described above.

### Retina explant cultures

E14 C57Bl/6 retinas were isolated and placed in matrigel as described above. Thalami-conditioned media or recombinant MHCI(1-298) (both described below) was then added to the indicated concentration. Control cultures received the same volume of conditioned media from wildtype thalami or COS cells (American Type Culture Collection, Manassas, VA) transfected with nonrecombinant plasmid. The cultures were coded and incubated further at 37°C in 5% CO^2^ for ≈48 hours. The experiment was terminated by replacing the media with 4% PFA and storing the slides at 4°C. Images of retinal explant cultures were imported into NIH Image Analysis software and analyzed for area covered by the neurite projections. Statistical significance was determined using a t-test or ANOVA.

### Production of and testing of thalami conditioned media

Wildtype or NSE-D^b^ thalami from 8–12 E14 fetuses were cultured for 3–5 days in Neurobasal media with L-glutamine, antibiotics and 1X B27 supplement. The conditioned media was harvested and added to fresh wildtype retina explants at a 1∶1 dilution with fresh media The cultures were incubated 2 days and scored in a blinded fashion as described above.

### COS cell aggregate and retina explants co-cultures

COS cells were transfected with a plasmid encoding the full-length D^b^ cDNA that contains MHCI's transmembrane and intracellular domain, or with a control plasmid (with an antisense D^b^ cDNA insert). COS cells were harvested 24 hours later and aggregates were made by hanging 10^5^ COS cells in a 20 µl drop for 24 hours. An aggregate was then placed ≈0.5–1 mm from a wildtype C57Bl/6 retina explant in a matrigel. After incubating 48 hours the cultures were fixed. In a blinded fashion, a microscopic field of view was scored for the number of neurites making contact with the COS cell pellet at multiple depths of focus.

### Production of sMHCI(1-298) from COS cells

A truncated MHCI-D^b^ cDNA (containing exons 1–4 (amino acids 1–298) plus a stop codon was subcloned into pIRES (Invitrogen). Due to the lack of a transmembrane spanning region, this MHCI molecule is secreted. COS cells were transfected (LipofectAMINE 2000, Invitrogen) with the parental plasmid, or the plasmid encoding sMHCI(1–298). The COS cell transfection efficiencies with the parental plasmid and the sMHCI(1–298) encoding plasmid were similar, as judged by the FACS analysis of GFP expression (which is also expressed by the pIRES plasmid). Forty eight hours later, the conditioned media was harvested and added to fresh explant cultures at the concentration indicated.

### Testing compounds that modulate cyclic nucleotide levels

cAMP (20 µM) or Rp-cAMPS (R-diasteriomer of adenosine 3′,5′-cyclic-phosphorothioate) (20 µM) a non-hydrolyzable antagonist of cAMP (Sigma-Aldrich) were added to the matrigel (prior to solidification) and the media of the explant cultures at the indicated concentrations. sMHC(1–298) was added to some wells to a final concentration of 500 pM. Control cultures received the same volume of conditioned media from nonrecombinant plasmid transfected COS cells. The cultures were coded and incubated at 37°C in 5% CO_2_ for ≈48 hours and analyzed in a blinded manner using NIH Image software for the area covered by the neurite projections and the data analyzed using t-test or ANOVA.

## Results

### Retina explant neurite outgrowth is stunted when in proximity to thalami that express elevated levels of neuronal MHCI

**Figure 1:**
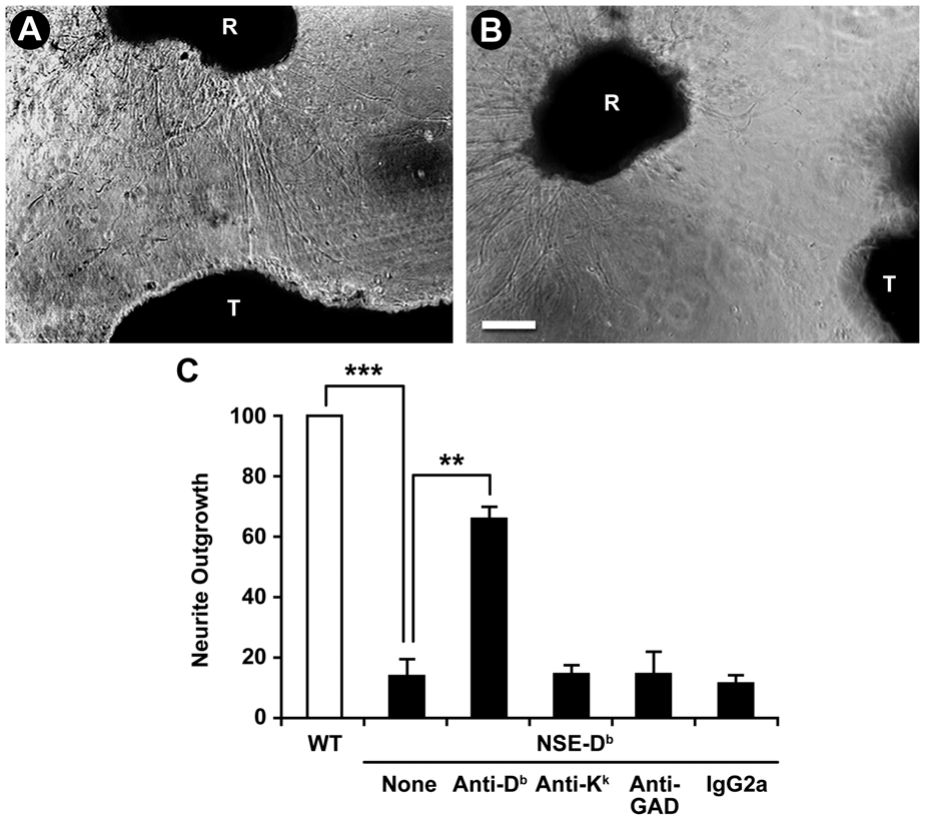
Retina explant neurite outgrowth in co-cultures with wildtype or NSE-D^b^ thalami. Representative images of wildtype E14 retina explant (labeled “R”) neurite outgrowth when cultured near to a wildtype (A) or NSE-D^b^ (B) E14 thalami (labeled “T”). White scale bar = 100 microns. (C) Group data of retinal neurite outgrowth toward a wildtype (wt) thalamus (open bar), or a NSE-D^b^ thalamus (black bars), in the absence, or presence, of different IgG2a antibodies. N = at least two co-cultures of each group were tested side-by-side in 4 separate experiments. **p<0.005, ***p<0.001 by Student's t-test.

We cultured wildtype C57Bl/6 retina explants a short distance from a wildtype C57Bl/6 or a NSE-D^b^ mouse thalamic explant. When juxtaposed with a wildtype thalamus explant, the retinas extended axon projections in all directions, and formed contacts with thalamic cells (representative image in Fig. 1A, group data in Fig. 1C). In contrast, retinas cultured near to a NSE-D^b^ thalamus extended much shorter processes on the side nearest to the thalamus (Figs. 1B, 1C). There was no reduction of the number of neurites, or turning of the projections away from the D^b^ thalamus as occurs in the presence of a diffusible repulsive factor–rather, neurite outgrowth was stunted on the side proximal to the NSE-D^b^ thalamus. These data suggest that NSE-D^b^ thalami release a diffusible factor(s) which can inhibit RGC neurite outgrowth at a distance.

### The neuroinhibitory factor from NSE-D^b^ thalami is soluble D^b^

Based on our previous finding that picomolar levels of recombinant MHCI monomers (but not heavy chain or β2M alone) inhibited RGC neurite outgrowth *in vitro* [17], we hypothesized that the diffusible inhibitory factor from NSE-D^b^ thalami was sMHCI. In wildtype mice, neuronal MHCI expression is induced postnatally in the thalamus during the period of activity-dependent remodeling [13]. In NSE-D^b^ mice however, the NSE promoter drives the ectopic expression of the D^b^ heavy chain in the embryonic CNS as cells differentiate into neurons. Previous studies of other tissues have found that increased MHCI expression leads to a concomitant increase in the release of sMHCI [22], [23], [24], [25]. To test whether the diffusible neuroinhibitory factor from NSE-D^b^ thalamic tissue was soluble D^b^ MHCI, we added a conformation-dependent anti-D^b^ mAb to the co-cultures and observed that this rescued the ability of retinas to extend processes towards a NSE-D^b^ thalamus (Fig. 1C). In contrast, addition of an isotype matched anti-K^k^ mAb, anti-GAD65 mAb or mouse IgG2a to the cultures failed to rescue neurite outgrowth toward the NSE-D^b^ thalamus (Fig. 1C). These data strongly suggest that the inhibitory factor from NSE-D^b^ thalami is conformationally correct sMHCI.

**Figure 2:**
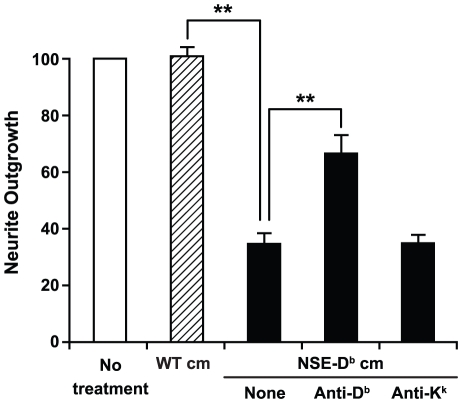
NSE-D^b^ thalami-conditioned media inhibits retina neurite outgrowth. Wildtype (wt) or NSE-D^b^ E14 thalamic explants were cultured and the conditioned media (cm) harvested. E14 retina explants were grown in fresh media (open bar), or a 1∶1 mixture of fresh media and cm from wt (hatched bar) or NSE-D^b^ (“NSE”, black bars) thalami, in the presence or absence of the indicated mAb, and the extent of neurite outgrowth assessed. N = at least two co-cultures of each group were tested side-by-side in 4 separate experiments. Data shown are the mean relative outgrowth ± SEM. **p<0.005 by Student's t-test.

We also cultured embryonic wildtype C57Bl/6 or NSE-D^b^ thalami and harvested the conditioned media from each. We then added the conditioned media to fresh retina explants in matrigel. Neurite outgrowth from retinas grown with conditioned media from wildtype thalamic cultures was similar to that from retinas cultured in fresh media (Fig. 2). In contrast, conditioned media from NSE-D^b^ thalamic cultures inhibited neurite outgrowth (Fig. 2). Addition of the conformation-dependent anti-D^b^-specific mAb, but not an anti-K^k^ mAb, to the conditioned media from NSE-D^b^ thalamic cultures significantly reduced its inhibitory activity (Fig. 2). These studies further indicate NSE-D^b^ thalami release sMHCI which can affect neurite outgrowth from other neurons.

### Aggregates of D^b^-expressing COS cells inhibit neurite outgrowth from nearby neurons

**Figure 3:**
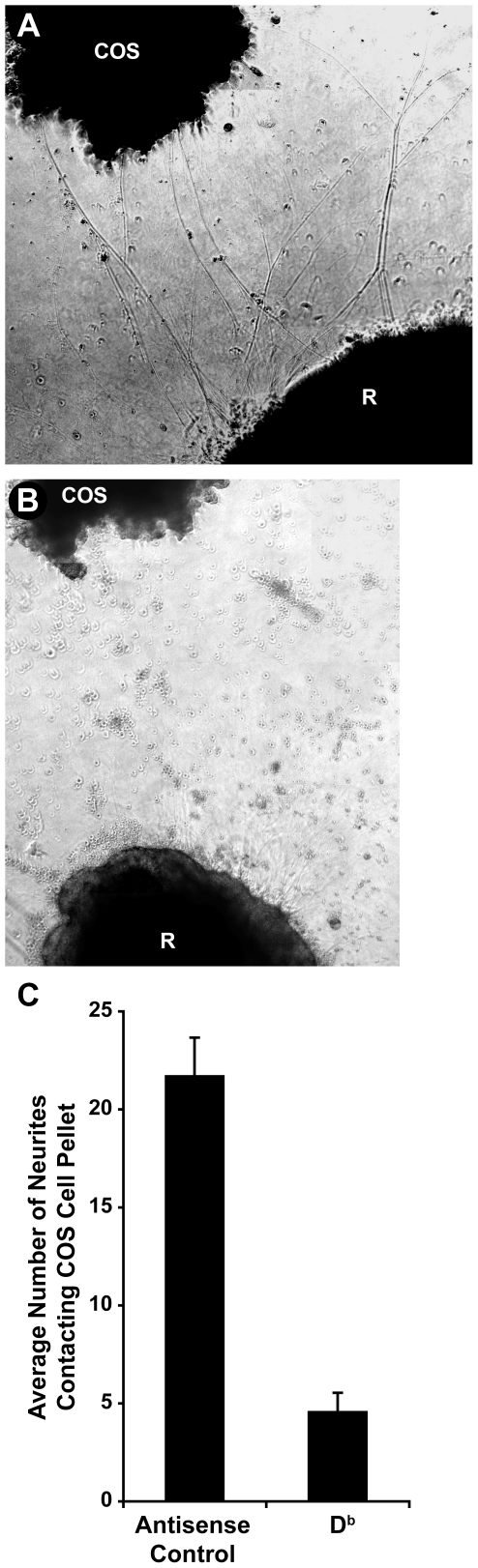
Aggregates of D^b^-expressing COS cells inhibit neurite outgrowth from nearby retinal explants. Representative images of neurite outgrowth from retina explant (labeled “R”) cultured near to an aggregate of control COS cells that were transfected with a plasmid which contains the Db cDNA in an antisense orientation (A), or express a full length Db cDNA and have conformationally correct D^b^ MHCI on their cell surface (B) (as demonstrated by FACS, data not shown). C) Graph of the average number of retina neurites contacting control or D^b^-expressing COS cell aggregate +/− SEM. N = 11–15 wells from 2 independent experiments. P ≤ 0.001 by Student's t test.

To further test the notion that MHCI-expressing cells can release sMHCI which can affect nearby neurons, we transfected monkey COS cells with a plasmid encoding a full-length D^b^ cDNA, or a control plasmid containing the D^b^ cDNA in the anti-sense orientation. The presence of membrane-bound D^b^ on the surface of experimental, but not control, transfected COS cells was confirmed by staining with anti-D^b^ mAb and FACS analysis (data not shown). The transfected cells were cultured using the hanging drop method to obtain an aggregate and the aggregate was placed ≈0.5–1 mm from a fresh E14 wildtype retina explant. Retinas that were cultured near to an aggregate of control COS cells sent out projections in all directions, some of which made contact with the COS cell aggregate (Fig. 3A). Retinas cultured near to a D^b^-expressing COS cell aggregate sent out a similar number of neurites, but few neurites made contact with the aggregate (Fig. 3B, 3C). The retina projections did not turn away from the aggregate, but rather had limited outgrowth from the region proximal to D^b^-expressing COS cells. Thus, COS cells that differ only in their expression of D^b^-MHCI have very different affects on neurite outgrowth from nearby neurons.

### Picomolar levels of sMHCI(1-298) from transfected COS cells inhibit retinal neurite outgrowth

**Figure 4:**
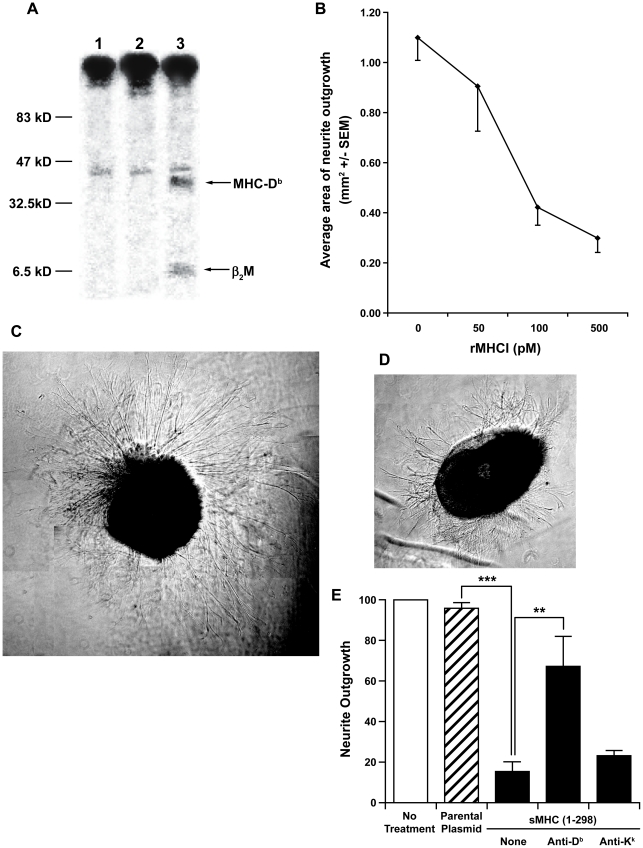
Recombinant, soluble MHCI [sMHCI(1-298)] inhibits retina neurite outgrowth. A) COS cells were transfected with a non-recombinant plasmid (lane 1) or one encoding D^b^ heavy chain amino acids 1–298 which lacks a membrane anchor (lanes 2 and 3), and grown for 48 hrs in ^35^S methionine containing media. The media was harvested, a conformation-dependent anti-MHCI-D^b^ (lanes 1 and 3) or anti-MHCI-K^k^ (lane 2) mAb was added, the bound proteins were collected on protein A sepharose beads and analyzed using SDS-PAGE and autoradiography. Image shows labeled immunoprecipitated proteins with the expected molecular weights of the D^b^ heavy chain and β2m in lane 3. B) Retina neurite outgrowth in the presence of a dose range of sMHCI(1–298). N = 8 wells from 2 experiments. There was a significant main effect of dilution concentration on average neurite outgrowth (F_(4,34)_ = 9.54; p<0.001). This was further confirmed by the systematic increase of neurite outgrowth with subsequent reductions in dilution concentration (R^2^ = 0.3215; F_(1,37)_ = 17.53; p<0.001). Representative composite images of an retina explant cultured with (C) conditioned media from control COS cell cultures, or (D) conditioned media from sMHCI(1–298) expressing COS cells (image shown is from a culture with 500 pM sMHCI(1–298)). Composite photos are shown at the same magnification. E) Average neurite outgrowth from retina explants grown in media alone (open bar), media to which conditioned media from COS cells transfected with a control plasmid (hatched bar), or a plasmid encoding D^b^(1–298) (black bars) was added. The indicated mAb was added to some cultures. N = 23–27 wells from 4 experiments. **p<0.01, ***p<0.002 by Student's t-test.

We engineered COS cells to produce sMHCI by transfecting them with a plasmid which directs the expression of a slightly truncated D^b^ (amino acids 1–298) which lacks its COOH-terminal membrane anchor, which we will refer to as sMHCI(1–298). We verified the presence of soluble D^b^ MHCI in the media from transfected COS cells by adding ^35^S methionine to some cultures and immunoprecipitating radiolabeled proteins in the culture supernatants with a conformation-dependent anti-D^b^ mAb followed by SDS-PAGE analysis (Fig. 4A). We detected D^b^ complexed with β2M in the conditioned media of COS cells transfected with the plasmid encoding D^b^, but not in those transfected with the control plasmid (Fig. 4A). The association of mouse D^b^ heavy chain with monkey β2M is expected since mouse heavy chains bind with greater affinity to human β2M than to mouse β2M, conferring greater stability to the complex [26]. ELISA analysis of conditioned media from unlabeled transfected COS cells indicated that D^b^ sMHCI(1–298) was present in the media at ≈ 1.0 nM. We measured retina explant neurite outgrowth in the presence of a dose range of sMHC(1–298) from transfected COS cells. We observed that the extent of inhibition by sMHCI(1–298) was concentration-dependent (Fig. 4B). Representative images of cultured retina explants are shown in Fig. 4C and 4D. The inhibitory effect of sMHCI(1–298) was detectable at ≈50–100 pM (Fig. 4B), a dose similar to that at which recombinant self-MHCI monomers began to have a discernable effect [17]. In a parallel study, we tested conditioned media from COS cells that express a truncated D^d^ heavy chain and observed that this had similar neuroinhibitory activity on retina explants from Balb/C (H-2^d^) mice (Fig. S1). Since the sMHCI(1–298) produced by COS cells are loaded with peptides from monkey cell proteins, and has similar effects as self-MHCI monomers, these observations provide further evidence that retina neuron MHCI receptors are not specific for the peptide presented by MHCI. The inhibitory effects of conditioned media from D^b^ sMHCI(1–298)-expressing COS cells could be largely prevented by subtracting the conditioned media with a conformation-dependent anti-D^b^ mAb, but not with an isotype matched MHCI-K^k^ specific mAb (4E), confirming that the neuroinhibitory effect was mediated by D^b^ sMHCI(1–298).

### A protein kinase A (PKA) antagonist neutralizes the neuroinhibitory effects of rMHCI

The adenylate cyclase-cAMP-protein kinase A pathway conveys an ‘off’ signal to both T cell and NK cell responses [27], [28]. We hypothesized that the inhibitory effects of sMHCI on neuronal outgrowth may also involve this signaling pathway. We therefore determined the effect of compounds that inhibit, or activate, a cyclic nucleotide-dependent pathway on neurite outgrowth from retina explants in the presence, or absence, of sMHCI(1–298).

**Figure 5:**
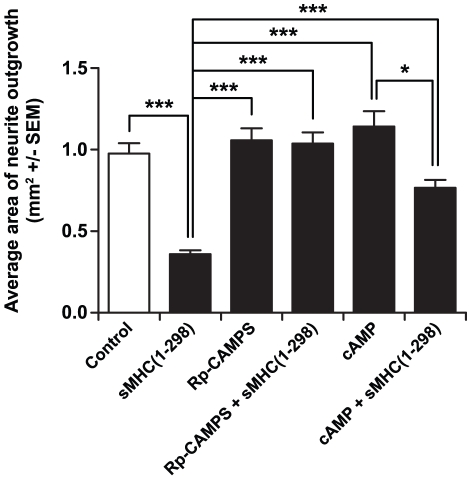
Modulation of intracellular cAMP levels affects sMHCI(1-298)-mediated inhibition of neurite outgrowth. Data shown are the average area of retina neurite outgrowth +/− SEM in the presence of media alone (control), sMHCI(1–298) (500 pM), the cAMP antagonist, Rp-cAMPS (20 µM), cAMP (20 µM), sMHCI(1–298)+Rp-cAMPS and sMHCI(1–298)+cAMP. There was a statistically significant difference across groups by ANOVA (p<0.001). N = 11–31 wells from 2–6 experiments. Statistical analyses were measured using ANOVA with post-hoc Tukey-Kramer multiple comparisons test. *p<0.05, ***p<0.001.

Retinas cultured in the presence of COS cell-produced sMHCI(1–298) (but no drug), had significantly less neurite extension compared to cultures without sMHCI(1–298) (Fig. 5). Neurite outgrowth from retinas cultured in the presence of Rp-cAMPS, a PKA inhibitor was similar to that of control retinas grown in media alone. Addition of Rp-cAMPS to retina explant cultures containing sMHCI(1–298) almost completely prevented the inhibitory effects of sMHCI(1–298) (Fig. 5), suggesting that part of the signaling cascade behind the inhibitory action of MHCI involves a cyclic nucleotide-dependent pathway. Retinas grown in the presence of cAMP had on average greater neurite outgrowth, as has been previously observed [29], but this was not statistically significant in our culture system. Addition of cAMP to retina cultures only partially prevented the inhibitory effects of sMHCI(1–298); the average neurite outgrowth in the presence of sMHCI(1–298) was significantly greater in the presence of cAMP, but this was still significantly less than that in cultures without sMHCI(1–298) (Fig. 5). These data suggest that the inhibitory effect of sMHCI is highly dependent on PKA and that this inhibition can be partially down-regulated by exogenous cAMP.

## Discussion

The elevated neuronal MHCI expression in NSE-D^b^ mice is associated with lower levels of synaptic markers and fewer neurons in some regions of their hippocampus, as well as a smaller area of contralateral retina projections in their dLGN [19]. The current studies were aimed at further understanding how elevated neuronal MHCI levels affect neuronal connections using an *in vitro* embryonic retina-thalamic co-culture system. In wildtype thalami, MHCI expression is up-regulated postnatally and is activity-dependent [13]. We observed that embryonic retina explants that were cultured near to a embryonic wildtype thalamus extended neurites which formed connections the thalami, as expected. In contrast, there was a conspicuous lack of neurite outgrowth from retina explant regions that were proximal to thalamic explants from NSE-D^b^ mice, suggesting that this thalamic tissue released a factor that inhibited nearby neurite outgrowth. Since our previous studies showed that recombinant MHCI monomers (but not heavy chain alone or β2M alone) inhibited neurite outgrowth *in vitro* [17], we surmised that the NSE-D^b^ thalami were producing sMHCI which inhibited neurite outgrowth from neighboring neurons. Indeed, in other tissues, upregulated MHCI expression leads to increased release of sMHCI [22], [23], [24], [25]. However, it was also possible that the transgene in some way reduced thalamic secretion of neurotrophic factors or induced the expression of a soluble inhibitory factor(s) other than sMHCI. We observed that neurite outgrowth could be rescued in large part by adding a conformation-dependent anti-D^b^ mAb to these cultures, pointing to diffusible conformationally correct D^b^ sMHCI as the inhibitory factor released from NSE-D^b^ thalami.

We also examined how retina neurites interacted with monkey COS cells that were programmed to express a full-length D^b^ cDNA. FACS analysis with a conformation-dependent mAb confirmed the presence of D^b^-MHCI on their cell surface. While retina neurites made contacts with a nearby COS cell aggregates transfected with a control plasmid, they had little neurite outgrowth from regions near to D^b^-expressing COS cell aggregates and made few contacts with these cells. These studies show that transfected cells that differ in their expression of a full-length mouse MHCI heavy chain cDNA differentially affect neurite outgrowth from nearby neurons.

Our studies with COS cell-produced sMHCI(1–298) showed that sMHCI(1–298) inhibited neurite outgrowth at 50–100 pM, a level similar to that of recombinant D^b^-MHCI monomers loaded with a particular mouse self-peptide [17]. The D^b^-sMHCI released from the transfected COS cells present a vast array of different peptides from monkey cell proteins. This provides another line of evidence indicating that RGC MHCI receptors are not sensitive to the peptide presented by MHCI. Because of this lack of specificity for the peptide presented, any conformationally correct sMHCI is a potential ligand, which may be why neurons are sensitive to such low levels of sMHCI. Notably, little or no sMHCI is detected in CSF of healthy individuals [30] indicating that the blood brain barrier effectively blocks sMHCI in peripheral blood from entering the CNS. Consequently, the level of MHCI expression in a particular brain region may be the major regulator of local sMHCI levels.

In the immune system, T cells that engage soluble MHCI, or membrane-bound MHCI without sufficient co-stimulatory signals, enter a state of inactivity (anergy) or can apoptose [31], [32], [33]. T cell inactivation by MHCI can be prevented by blocking the T cell receptor's signaling cascade with an antagonist (Rp-cAMPS) of cAMP-dependent PKA [28], [34]. Similarly, sMHCI down-regulates NK cell function or induces NK cell apoptosis [35], [36], and compounds that elevate cAMP suppress both NK and dendritic cell function [37]. We found that Rp-cAMPS itself had no discernable effect on retinal neurite outgrowth *in vitro.* However, its inclusion in cultures containing sMHCI(1–298) prevented the inhibitory effects of sMHCI(1–298) on neurite outgrowth. Evidently, sMHCI can inhibit cellular functions by pathways that involve PKA in both the nervous and immune systems. Conceivably, the neuroinhibitory activity of sMHCI that we have observed may be a neurological counterpart of its inhibitory action on NK cells or T cells that engage sMHCI or membrane-bound MHCI in the absence of co-stimulatory signals.

Our observations concerning sMHCI, together with the abnormalities observed in NSE-D^b^ mouse neurodevelopment [19], suggest several new possibilities concerning MHCI's neurobiological activities. Neuronal MHCI expression in the thalamus peaks during the period of activity-dependent remodeling [13]. Studies with other tissues have shown that increased MHCI expression is accompanied by increased release of sMHCI [22], [23], [24], [25]. This could be due to increased shedding of intact MHCI, increased heavy chain transcription leading a concomitant increase in alternatively spliced transcripts and/or more substrate for the action of proteases. After appropriate connections are formed, elevated membrane-bound MHCI and sMHCI levels may help limit axon outgrowth into that region and additional synaptogenesis. Indeed, we have observed that the NSE-D^b^ mice have reduced synaptic markers in some hippocampal regions and are very deficient in compensatory neuronal sprouting responses after a hippocampal lesion [19]. The sMHCI might bind to neuronal MHCI receptors in ways that activate receptor signaling, or conversely, prevent the MHCI receptor from binding to membrane-bound MHCI. Additionally, MHCI receptor interaction with sMHCI without the co-stimulatory signals that accompany MHCI receptor interaction with membrane-bound MHCI, may lead to different biological outcomes. Alternatively, like some other axon guidance molecules, MHCI and sMHCI may have bi-functional effects, exerting either negative or positive influences on axon growth depending on the context (e.g., their concentration, the levels of cyclic nucleotides, and the stage in development) [38], [39], [40], [41], [42].

Interestingly, mice deficient in complement protein C1q or C3 have impairments in retinogeniculate synapse elimination, similar to that seen in MHCI-deficient mice [16], suggesting that complement acts to tag supernumerary synapses for removal by phagocytotic mechanisms. Complement proteins can be synthesized by neurons and glia and their expression peaks during activity dependent remodeling [16]. C1q combines with serine esterases C1r and C1s to form the C1 protein. Notably, C1 can associate with β2M and its serine esterase activity can cleave MHCI between its α2 and α3 domains, releasing a biologically active sMHCI consisting of the α1 and α2 domains associated with β2M [43], [44], [45], [46], [47]. C3 also contributes to the formation of proteases. Both C1q expression and neuronal MHCI expression peak in the LGN during activity-dependent remodeling [13], [16]. The concurrent upregulation of both MHCI and complement protein expression could synergistically elevate sMHCI levels in the microenvironment which we have shown can act in *trans* to inhibit neurite outgrowth ([17], [48] and herein). Such complement cleavage would also reduce MHCI *cis*-associations with other molecules on the same cell surface. Additionally, MHCI molecules can dissociate, leaving free heavy chains on the cell surface which can associate in *cis* with other free heavy chains and receptors on the same cell surface and modulate cellular functions (reviewed in [49]). Complement cleavage of free heavy chains could reduce these *cis*-associations as well. Importantly, our recent studies suggest that the extent of MHCI *cis* interactions on the neuronal cell surface modulate axon outgrowth and polarity (Tina Bilousova and DLK, manuscript in preparation). Cleavage also leaves behind MHCI's α3 domain which can interact with some classical MHCI receptors of the innate immune system [50], [51], [52]. Further studies are needed to address these possibilities, but are hampered by the lack of nucleic acid probes and antibodies that can distinguish sMHCI and membrane-bound MHCI in tissue sections.

Neuronal MHCI and complement protein expression are upregulated in response to various insults (e.g. seizures, injury, cytokines and other inflammatory signals) [16], [53], [54], [55], [56], [57], [58], [59], [60], [61]. Our results in the current study, together with our previous observations of abnormalities in neuronal connections and neurorepair responses in the NSE-D^b^ mouse CNS suggest that such insults may increase local levels of MHCI and sMHCI which could affect neurodevelopment and neurorepair responses. It is of interest that the HLA region has been genetically associated with schizophrenia and autism in humans [62], [63]. If MHCI and/or sMHCI are found to have potential deleterious neurological effects, MHCI antibodies, soluble MHCI receptors or anti-inflammatory medications may useful to block these effects.

## Supporting Information

Fig. S1 **Data shows average neurite outgrowth from E14 Balb/C retina explants grown in media to which conditioned media from COS cells transfected with a control plasmid (hatched bar) or a plasmid encoding a truncated D^d^ heavy chain (black bar) was added.** N = at least 21 wells from 3 experiments. **p<0.001 by Student's t-test.
(EPS)
